# Ice-Binding Proteins Associated with an Antarctic Cyanobacterium, *Nostoc* sp. HG1

**DOI:** 10.1128/AEM.02499-20

**Published:** 2021-01-04

**Authors:** James A. Raymond, Michael G. Janech, Marco Mangiagalli

**Affiliations:** a School of Life Sciences, University of Nevada Las Vegas, Las Vegas, Nevada, USA; b Department of Medicine, Medical University of South Carolina, Charleston, South Carolina, USA; c Department of Biology, College of Charleston, Charleston, South Carolina, USA; d Department of Biotechnology and Biosciences, University of Milan—Bicocca, Milan, Italy; Kyoto University

**Keywords:** Antarctica, *Nostoc*, PEP C-terminal signal, horizontal gene transfer, ice-binding proteins

## Abstract

Ice-binding proteins (IBPs) have been identified in numerous polar algae and bacteria, but so far not in any cyanobacteria, despite the abundance of cyanobacteria in polar regions. We previously reported strong IBP activity associated with an Antarctic *Nostoc* species. In this study, to identify the proteins responsible, as well as elucidate their origin, we sequenced the DNA of an environmental sample of this species, designated *Nostoc* sp. HG1, and its bacterial community and attempted to identify IBPs by looking for known IBPs in the metagenome and by looking for novel IBPs by tandem mass spectrometry (MS/MS) proteomics analyses of ice affinity-purified proteins. The metagenome contained over 116 DUF3494-type IBP genes, the most common type of IBP identified so far. One of the IBPs could be confidently assigned to *Nostoc*, while the others could be attributed to diverse bacteria, which, surprisingly, accounted for the great majority of the metagenome. Recombinant *Nostoc* IBPs (nIBPs) had strong ice-structuring activities, and their circular dichroism spectra were consistent with the secondary structure of a DUF3494-type IBP. nIBP is unusual in that it is the only IBP identified so far to have a PEP (amino acid motif) C-terminal signal, a signal that has been associated with anchoring to the outer cell membrane. These results suggest that the observed IBP activity of *Nostoc* sp. HG1 was due to a combination of endogenous and exogenous IBPs. Amino acid and nucleotide sequence analyses of nIBP raise the possibility that it was acquired from a planctomycete.

**IMPORTANCE** The horizontal transfer of genes encoding ice-binding proteins (IBPs), proteins that confer freeze-thaw tolerance, has allowed many microorganisms to expand their ranges into polar regions. One group of microorganisms for which nothing is known about its IBPs is cyanobacteria. In this study, we identified a cyanobacterial IBP and showed that it was likely acquired from another bacterium, probably a planctomycete. We also showed that a consortium of IBP-producing bacteria living with the *Nostoc* contribute to its IBP activity.

## Abstract (precis)

The horizontal transfer of genes encoding ice-binding proteins (IBPs), proteins that confer freeze-thaw tolerance, has allowed many microorganisms to expand their ranges into polar regions. One group of microorganisms for which nothing is known about its IBPs is cyanobacteria. In this study, we identified a cyanobacterial IBP and showed that it was likely acquired from another bacterium, probably a planctomycete. We also showed that a consortium of IBP-producing bacteria living with the *Nostoc* contribute to its IBP activity.

## INTRODUCTION

Many microorganisms in icy environments make ice-binding proteins (IBPs) to mitigate the effects of freezing and thawing (1, 2). Unlike the antifreeze proteins (AFPs) of fish and insects that are present in milligram-per-milliliter concentrations and that actually lower the freezing point (3), microorganismal IBPs are present at the microgram-per-milliliter level and have a negligible effect on the freezing point. However, they have other effects on ice that are clearly important to microorganisms. They protect against freeze-thaw damage through their powerful ability to prevent the recrystallization of ice, which is considered damaging to cell walls (1). They can also preserve a liquid environment for organisms living in ice. In sea ice, they change the structure of the ice in a way that hinders the drainage of trapped water (4, 5), and in glacier ice, they preserve a thin layer of water between ice grains by preventing the ice grains from recrystallizing (6). Finally, they also provide a means of attaching cells to an ice substrate in a way that might enhance their access to sunlight or nutrients (7, 8).

In addition, most IBPs are secreted and act extracellularly, whereas AFPs act internally. The most common microorganismal IBPs have similar structures based on an ∼200-amino-acid domain referred to as DUF3494; they are found in bacteria, archaea, algae, and fungi (for examples, see references 9 and 10). Other, less common IBPs have been identified in a chlorophyte alga (11–13) and a bacterium (7). A central question concerns the origin of IBPs, as their phylogeny over a broad array of taxa appears unrelated to the phylogeny of the host organisms. This strongly suggests that the IBP genes were acquired by horizontal gene transfer (HGT) (14, 15). In the case of unicellular algae, HGT probably enabled their spread into polar and alpine regions, as all ice-associated algae that have been examined so far produce IBPs, while mesophilic algae do not (for example, see reference 2). However, little is known about the donor organisms or the mechanisms and timing of transfer. One approach to answering these questions is to identify IBPs in previously unexplored taxa to make it easier to recognize patterns in their distribution. One group of polar microorganisms for which no IBP had been identified at the time this study began (in 2016) is the cyanobacteria. At that time, the more than 200 cyanobacterial genomes in the databases contained no proteins resembling known IBPs. However, strong IBP-like activity was found in a species of *Nostoc* from a glacial melt stream in the McMurdo Dry Valleys of Antarctica (16), a habitat in which the microbial community is dominated by *Nostoc* (17). To identify the source of activity, we sequenced the genomic DNA of an IBP-active sample of *Nostoc* and its associated microorganisms and attempted to identify IBPs by looking for known IBPs in the metagenome and by tandem mass spectrometry (MS/MS) proteomics analysis of ice-affinity purified proteins. Here, we show that the Dry Valley *Nostoc* has a DUF3494-type IBP that appears to have been acquired by horizontal gene transfer. Furthermore, many other DUF3494-type IBPs from a consortium of surface bacteria appear to contribute to its IBP activity.

## RESULTS

### Sample collection site.

**FIG 1:**
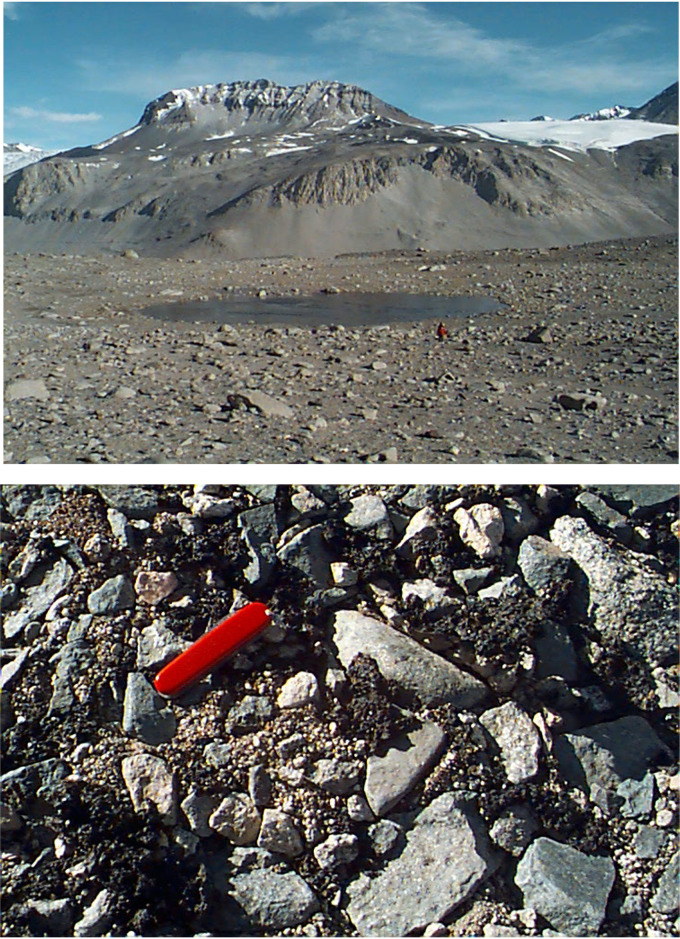
*Nostoc* collection site in the Taylor Valley, Antarctica, in January 2000. (Top) A view of the region. Kukri Hills are in the background, and the upper portion of the Hughes Glacier is visible on the left. A team member (red coat) provides scale. (Bottom) Clumps of dried *Nostoc* in this region.

*Nostoc* samples were collected from rocky surfaces in the Taylor Valley near the base of the Hughes Glacier (77°44′S, 162°28′E) (Fig. 1, top). The samples were in the form of desiccated, black leafy clumps (Fig. 1, bottom).

### 16S and 18S analyses of the metagenome.

**FIG 2:**
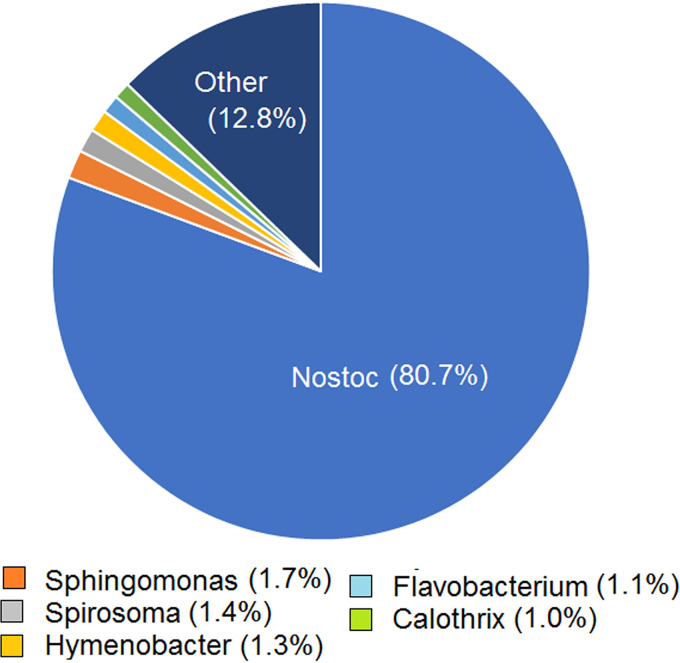
Major bacterial species in the *Nostoc* metagenome based on the frequency of 16S rRNAs in 35,791 reads with matches to named species and E values of <1e−30.

A search of the *Nostoc* metagenome for bacterial 16S rRNA sequences yielded 35,791 reads with E values of <1e−30 that could be assigned to 359 genera representing 458 named bacterial species. The major genera based on the frequency of the 16S reads were, in decreasing order, *Nostoc*, *Sphingomonas*, *Spirosoma*, *Hymenobacter*, *Flavobacterium*, and the cyanobacterium *Calothrix* (Fig. 2). The *Nostoc* reads could all be assigned to one 16S sequence (GenBank accession no. MN081603). We designated our species *Nostoc* sp. HG1 after its location near the Hughes Glacier. The closest match (99.9%) was the 16S sequence of Antarctic *Nostoc* sp. ANT.L52B.8 (AY493593), which was collected in a small coastal lake on the other side of the continent (Appendix 1 of reference 18). However, this species appeared to differ from HG1 (see below).

The contigs assembled from the metagenome reads had a total length of about 616 million bp. Because the *Nostoc* genomes in GenBank have sizes of only 8 to 9 Mbp, almost all of the contigs appeared to belong to the many bacteria identified in the 16S analysis. This was surprising because the *Nostoc* tissue appeared to account for almost all of the biomass of the sample.

The metagenome also contained a few eukaryotic 18S rRNA sequences. The most prominent was an 18S sequence (contig 1236) nearly identical (99.3%) to that of the cosmopolitan rotifer Adineta vaga (GenBank accession no. GQ398061). A. vaga has a genome size of 289 Mb, but it appeared to account for only a small fraction of the contig file: a tblastn search using the 731 *A. vaga* proteins in the NCBI database as queries and an E value cutoff of 1e−100 yielded 152 contigs with a total length of only 981 kb. Another 18S sequence (contig 82900) was attributed to an ascomycete fungus, the closest match (96.1%) being a leaf fungus, Guignardia alliacea (GenBank accession no. NG_064827). A third 18S sequence (contig78065) was 98.2% identical to that of an alveolate-related eukaryote from the periglacial environment at the top of Mt. Kilimanjaro (GenBank accession no. KX771895). Several reads identical to the 18S rRNA of the Antarctic tardigrade Acutuncus antarcticus (GenBank accession no. AB753790) were also found. (This was confirmed by observations of the revival of several tardigrades in the *Nostoc* sample after 20 years at −25°C.) No 18S sequences matching those of nematodes, tardigrades, or mites that are known to inhabit the same region (McMurdo Dry Valleys) were found in the metagenome. In many regions, including Antarctica (19), *Nostoc* is a symbiont of the lichen *Peltigera*. However, no *Peltigera* 18S sequences were found in the metagenome and the sample showed no signs of the presence of a lichen.

### IBP genes.

A tblastn search of the contig file for DUF3494-type IBP genes yielded 116 contigs encoding proteins that matched DUF3494 protein sequences in GenBank with E values <1e−20. Among them, 25 complete or nearly complete genes could be assembled, several of which had two DUF3494 domains (Table S2). All of them had bacterial IBPs as their closest matches. Signal peptides were present in 23 of the sequences and were ambiguous in the other 2, suggesting that the IBPs are secreted from the cells. These results indicate that the *Nostoc* metagenome is rich in IBP-bearing bacteria.

Initially, none of the IBP genes in the *Nostoc* metagenome could be unambiguously attributed to *Nostoc*. Searches for other types of IBP, including plant antifreeze proteins and bacterial ice-nucleating proteins, were unsuccessful. We then tried a proteomics approach, attempting to identify peptides of digested ice-affinity-purified proteins by mass spectrometry and matching them to sequences in the metagenome. None of the proteins identified matched known IBPs. Two proteins that were prominent in the MS results and that were confirmed to be *Nostoc* proteins by their sequences were phycocyanin, a cyanobacterial pigment, and fasciclin, a protein involved in cell adhesion. Both are highly water soluble. However, solutions of *Spirulina* C-phycocyanin (Sigma) and recombinant *Nostoc* fasciclin (GenScript; otherwise not described) showed no activity.

Subsequently, the genomes of three *Nostoc* species, from Iceland, Canada, and Japan, with very similar (98% identical) 16S sequences were submitted to GenBank (Table S3). Each was identified as a symbiont of the lichen *Peltigera*, a foliose lichen whose lobes are underlaid by a layer of *Nostoc*. Furthermore, each had a single gene encoding a DUF3494-type protein. The reason for sequencing two of the genomes was to better understand whether specific sets of genes are associated with a stable symbiosis (20), although IBP genes were not mentioned in that study. The accessions (Table S3), which appeared to have been annotated automatically, were not identified as ice-binding proteins. Two were identified as DUF3494-containing proteins and one was identified as a hypothetical protein. A gene very similar to the lichen symbiont IBPs was found in a contig from the *Nostoc* metagenome, and it could be unambiguously attributed to *Nostoc* sp. HG1 because it closely matched the IBP genes and downstream intergenic spaces and flanking genes in each of the three symbiont species. We thus designated this gene nIBP (GenBank accession no. MN082380). The three *Nostoc* symbiont IBPs are also by far the closest matches to nIBP in the databases. As mentioned above, we found no evidence that *Nostoc* sp. HG1 was associated with a lichen. Furthermore, *Nostoc* ANT.L52B.8 (the Antarctic *Nostoc* species identified above as having an 16S rRNA sequence nearly identical to that of HG1) appears to be a different species, as several PCR primer pairs that worked on HG1 IBP did not yield products from ANT.L52B.8 DNA. (The integrity of the latter DNA was confirmed by its production of a PCR product of the expected size from HG1 16S primers.)

**FIG 3:**
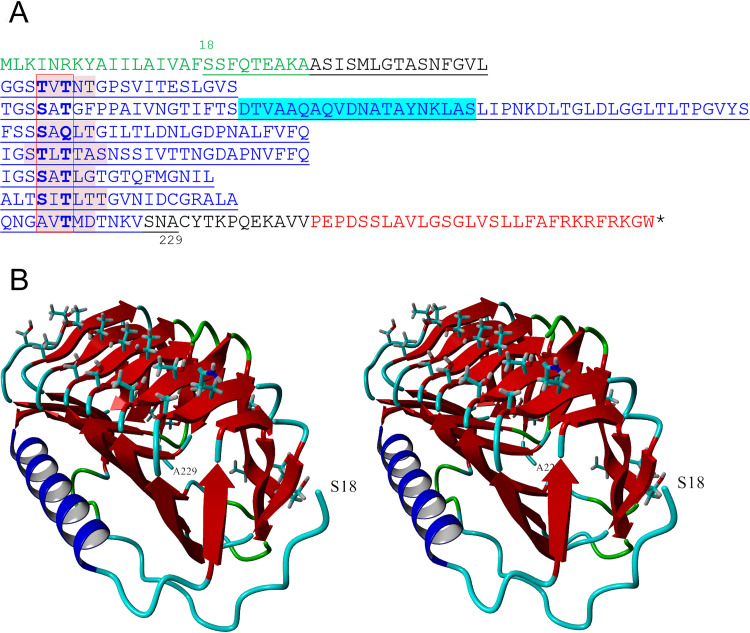
*Nostoc* IBP sequence and structure. (A) IBP sequence. The sequence consists of an N-terminal signal peptide (green), a DUF3494 domain (blue), and a C-terminal PEP C-term signal (red). The underlined region from S18 to A229 is the portion of the molecule modeled in panel B. Blue highlight, alpha helix; red highlight, beta strands on predicted ice-binding site. The red box shows alignment of Thr and Ser residues (bold) that are predicted to interact with ice. (B) Stereoviews of the predicted structure, in which the ice-binding site is on top.

The sequence of nIBP (Fig. 3A) is typical of other algal and bacterial IBPs in having an N-terminal signal peptide (green letters) followed by a DUF3494 ice-binding domain (blue letters). The signal peptide usually means that the protein is secreted from the cell, after cleavage of the signal peptide. The DUF3494 domain has been arranged to reflect the repeating units in the predicted three-dimensional (3D) structure (Fig. 3B). It includes seven beta sheets (red) that are rich in serine and threonine residues (boxed) and an alpha helical region (blue).

Interestingly, nIBPs (as well as its three *Peltigera* symbiont homologs) differ from other reported DUF3494 IBPs in that they also have a C-terminal sorting signal called PEP-Cterm (red letters in Fig. 3A), where the amino acid motif PEP is followed by a hydrophobic membrane-spanning domain and a highly basic region just before the C terminus. The function of this domain is discussed below.

### 3D structure of nIBP.

The structure of nIBP, predicted from the structure of an IBP from a bacterial consortium of the ciliate *Euplotes focardii* (*Efc*IBP), is a beta solenoid in the form of a triangular prism formed by three different sides (a, b, and c) (Fig. 3B and Fig. S2A), similar to the structures of other DUF3494 proteins determined by X-ray crystallography (see Discussion). nIBP shares high sequence identity (>43%) with *Efc*IBP and an IBP from the Antarctic fungus *Antarctomyces psychrotrophicus* (*Anp*IBP), which are characterized by the absence of capping head region at the top of the solenoid (21, 22). In most other DUF3494 proteins whose structures have been determined, the b side was identified as the ice-binding site because of the presence of an orderly array of outward-pointing hydrophilic side chains and because amino acid substitutions on this side decreased activity (9, 10). The b side of nIBP, like that of its template *Efc*IBP and its homologous *Anp*IBP, has two prominent rows of hydrophilic side chains (mostly threonine and serine) (Fig. S2B). The average distance between the side chains on adjacent coils ranges from 4.3 to 4.9 Å (Table S4), depending on which atoms on the side chains are selected. These distances are close to the repeat distance along the ice *a* axis (4.52 Å).

### Recombinant *Nostoc* IBPs.

**FIG 4:**
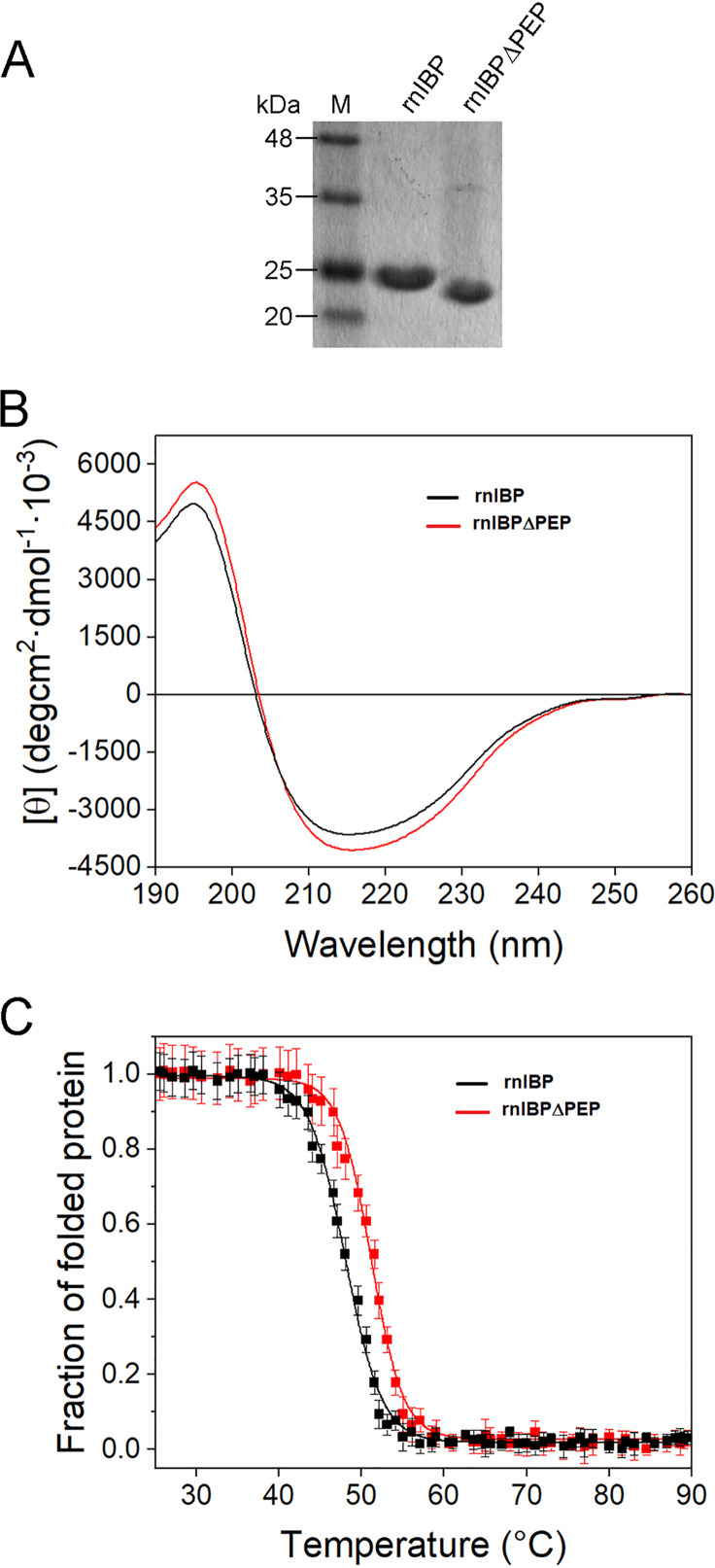
Molecular masses and circular dichroism properties of recombinant IBPs rnIBP and rnIBPΔPEP. (A) SDS-PAGE of purified rnIBP (with PEP-Cterm signal) and rnIBPΔPEP (after removing SUMO tag). M, molecular mass markers. Each lane contains ∼5 μg of purified protein. (B) UV CD spectra at concentrations of 5 μM in phosphate buffer. (C) Thermal stability as shown by the effect of temperature on the ellipticity at 204 nm. The initial CD signals were normalized to 1. Error bars indicate standard deviations from three independent experiments.

The recombinant IBPs, one with the PEP domain (rnIBP) and one without the PEP domain (rnIBPΔPEP), were produced in Escherichia coli BL21(DE3) as detailed in the supplemental material. The molecular weights of rnIBP and rnIBPΔPEP as shown by SDS-PAGE analyses were ∼25 kDa and ∼23 kDa, respectively (Fig. 4A), which are in agreement with their predicted molecular weights of 25,640 and 21,402 Da, respectively.

The circular dichroism (CD) spectra of rnIBP and rnIBPΔPEP (Fig. 4A) present typical profiles of a protein rich in beta sheet structures, with negative bands at ∼212 nm and positive ellipticity at ∼195 nm, in agreement with the predicted secondary structure in Fig. 3. Above 40°C, the ellipticity signal was rapidly and irreversibly lost, with a transition midpoint at 47.9 ± 0.7°C (Fig. 4B), similar to that for the recombinant IBP from the ice bacterium Flavobacterium frigoris (23).

### Ice-binding activity.

**FIG 5:**
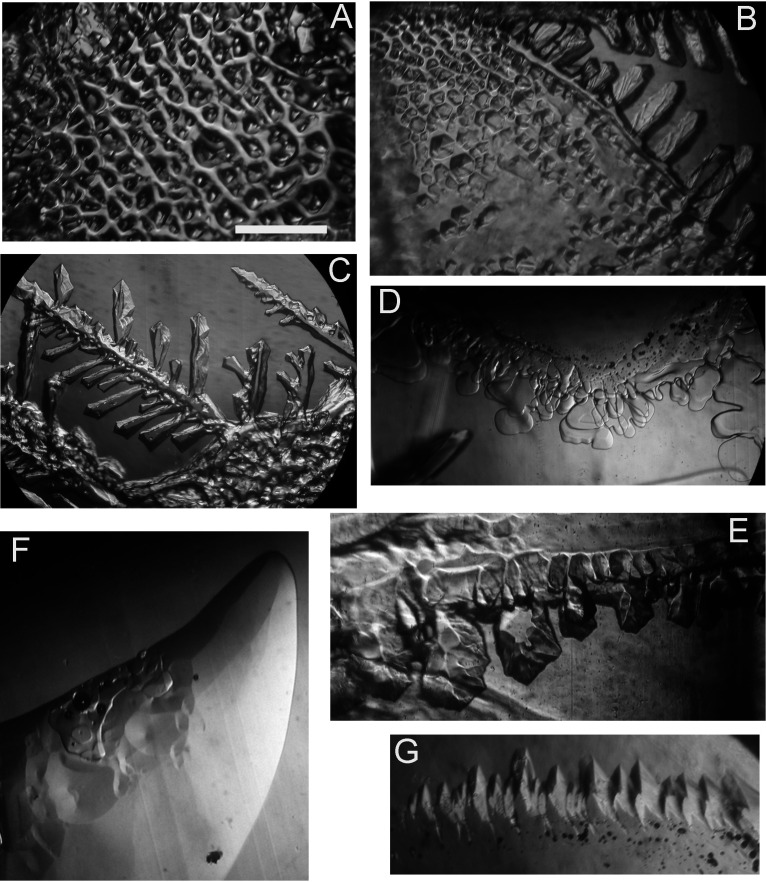
Ice-binding activities of nonaxenic and recombinant *Nostoc* sp. HG1 samples. Highly distorted surfaces of a growing seed crystal are indications of the presence of ice-binding proteins. (A) Concentrated rinse water, indicating that at least some of the activity resides on the surface of the cells. (B and C) Supernatants of homogenized rinsed (B) and unrinsed (C) *Nostoc* tissue. The activities in panels A to C are probably mostly due to IBPs produced by *Nostoc*’s microbial community. (D and E) Recombinant nIBP samples (rnIBP), including one that failed to solubilize (D) and one that was partially solubilized by detergent and shaking (E). (F and G) Recombinant rnIBPΔPEP samples showing shallow pitting (F) and highly faceted edges that formed after another 30 min of growth (G). Scale bar, 1 mm. The scales in all panels are approximately the same.

A 1998 sample of *Nostoc* from the base of the Hughes Glacier was previously reported to have strong ice-binding activity (Fig. 1b in reference 16), although the ice-binding molecules were not identified and the possibility that they came from other organisms associated with the *Nostoc* could not be ruled out. Figure 5 shows additional examples of the activity assayed recently with the 2000 sample. A solution of water-soluble material rinsed from the surface of a clump of *Nostoc* cells and then concentrated by freeze-drying was very active, as shown by the highly distorted growth (Fig. 5A). Supernatants of homogenates of rinsed cells (Fig. 5B) and unrinsed cells (Fig. 5C) were also active, as shown by pitting on the basal plane of ice and highly faceted dendrites growing from the prism faces of the ice. These results demonstrate the presence of IBP activity in both the environmental sample as a whole and on its surface. Because the nIBP gene represents only a tiny fraction of the IBP genes in the sample and because the rinse water is highly active, it is likely that most of the activity is due to noncyanobacterial bacteria (see also Discussion below).

The recombinant *Nostoc* IBP samples (rnIBP and rnIBPΔPEP), which had the appearance of a glass after freeze-drying for shipment, were poorly soluble. Initially, the rnIBP samples showed no activity (Fig. 5D). However, one of them showed strong ice-structuring activity (development of facets on the prism faces of ice and pitting on the edge of the basal plane) after being treated with detergent and shaking (see Materials and Methods) (Fig. 5E). The other recombinant, rnIBPΔPEP, caused the formation of shallow circular pits (Fig. 5F) and highly structured facets (Fig. 5G).

### IBP closest matches.

The closest matches to nIBP in GenBank were bacterial IBPs, while matches to fungal and plant IBPs had much lower E values. The closest match to nIBP (other than the IBPs of the *Peltigera* symbionts) was a DUF3494 protein from the planctomycete Singulisphaera acidiphila DSM 18658, which was isolated from a peat bog in northern Russia. The identities over the DUF3494 domain at the amino acid and nucleotide sequence levels (69% and 72%, respectively) are high compared to the identities reported for other IBP pairs. nIBP also appears to differ in nucleotide composition from other *Nostoc* genes: the GC content of its DUF3494 domain (52.9%) is 7 standard deviations (SDs) above the GC content of 20 randomly selected *Nostoc*-specific contigs (41.4% ± 1.6% [mean ± SD]) and closer to the GC contents of *S. acidiphila* IBP (61.1%) and the *S. acidiphila* genome (62.16%) (24). Interestingly, the bacterial IBP gene in the contig file that was closest to nIBP (contig 740803; 62.6% amino acid identity) also appeared to be a planctomycete gene based on its closest match and high GC content (59.8%). On the other hand, nIBP has low similarities (<35%) to DUF3494 genes (NCBI accession no. PKL76429, PKL75809, and PKL75521) from a bacterium that is considered a close relative of the cyanobacteria (“*Candidatus* Melainabacterium”) (25).

## DISCUSSION

Here, we have described the first ice-binding protein in a cyanobacterium. Of more than 400 cyanobacterial genomes that have been sequenced so far, the Antarctic *Nostoc* sp. HG1 is the only one in which an active IBP (nIBP) has been confirmed. Similar genes have been found in the recently reported genomes of closely related *Nostoc* species in the Northern Hemisphere, although these species, unlike HG1, are all endosymbionts of the lichen *Peltigera*. These genes all encode DUF3494-type IBPs similar to those in many bacteria, fungi, and unicellular algae (1) that protect cells in various ways, including inhibiting the recrystallization of ice, preserving a liquid environment, and anchoring cells to a substrate (see the introduction). In the case of HG1, the IBPs appear to be anchored to the outer cell wall through their PEP-Cterm signal. Such proteins might have a greater effect at preventing ice recrystallization at the cell surface than would more distant secreted proteins. It seems less likely that nIBP has a role in anchoring HG1 to an icy substrate, as it appears to grow in shallow puddles and pools fed by melting glaciers where sunlight is plentiful.

However, *Nostoc* sp. HG1 does not appear to depend on nIBP alone, as it is covered with bacteria, 25 species of which have complete or nearly complete IBP genes and about 90 others whose genes could be partially assembled. Almost all of the complete IBP genes encode signal peptides, which indicates that the proteins are probably secreted, as is indicated by the high activity of rinse water (Fig. 5A). Additional evidence for strong bacterial IBP activity comes from a moss from the same region of Antarctica (26). The moss, like *Nostoc* HG1, has many species of bacteria on its surface that have DUF3494-type IBP genes. Also like HG1, the moss had very high IBP activity despite not having any identifiable IBP genes of its own, thus demonstrating that the surface bacteria have high IBP activity. In fact, IBP-bearing bacteria appear to be enriched in polar regions, as the frequency of their occurrence was found to be 1 or 2 orders of magnitude higher in the moss metagenome and in a sea ice metagenome than in mesophilic metagenomes (26).

DUF3494-type proteins are found in habitats worldwide, including the tropics and hot springs, which suggests that these proteins are designed to bind to a variety of substrates. A consensus IBP made up of 172 IBPs has a similar array of hydrophilic residues on one side of the protein (27), which suggests that, in general, these proteins are designed to bind to a substrate with a repeating unit, such as a mineral. A slight modification of the array could thus result in an affinity for ice and an ability to affect its growth, properties that would have aided the spread of microorganisms into regions exposed to freezing conditions.

**FIG 6:**
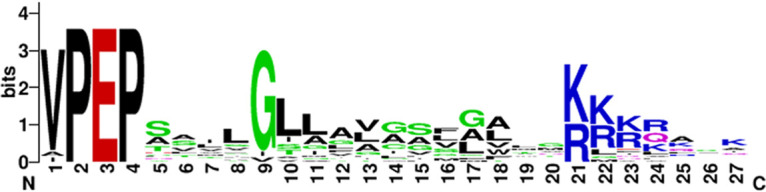
Logo diagram of 20 PEP-Cterm proteins found in the *Nostoc* HG1 genome.

nIBP and its *Peltigera* symbiont homologs are unusual in that they are the only IBPs so far reported to have PEP-Cterm signals. However, a search of GenBank for bacterial proteins with the annotations DUF3494 and PEP-Cterm yielded at least 23 such proteins. PEP-Cterm signals are found only in bacteria, and in the bacteria in which they are found, the genome usually contains 10 to 20 such proteins (28). Accordingly, a search of the *Nostoc* contig file yielded 20 additional proteins with PEP-Cterm sorting signals that could be attributed to *Nostoc* sp. HG1 (and not other bacteria in the metagenome). A logo diagram of the PEP-Cterm signals of these proteins clearly shows the three conserved parts of the signal (Fig. 6).

PEP-Cterm proteins are thought to be anchored to the outer membrane through a middle transmembrane domain, and they are typically found in species that secrete extracellular polymeric substances (EPSs). *Nostoc* species are well known for this characteristic, which may contribute to their noted ability to tolerate desiccation (29). PEP-Cterm proteins are suspected of being glycosylated and thus contributing to the EPSs (28). nIBP has several potential N- and O-linked glycosylation sites according to GlycoPP, but it is unknown whether it is glycosylated. Bacteria with a cohort of PEP-Cterm proteins have a membrane protein called exopolysaccharide locus H (EpsH) that along with a PEP-Cterm protein forms a protein export sorting system (28). The EpsH homolog gene is usually found among extracellular polysaccharide biosynthesis genes, including that for a histidine kinase (28). This is also the case in *Nostoc* sp. HG1, in which all of these genes are present in a contiguous 13.7-kb sequence (Table S5). Little is known about the functions of PEP-Cterm proteins, and BLAST searches of the 20 other *Nostoc* PEP-Cterm proteins did not reveal any specific functions. In fact, nIBP appears to be one of the first PEP-Cterm proteins to have a known function.

The PEP-Cterm signal on nIBP might help to explain our failure to observe nIBP in the mass spectrometry analyses. We assumed that the protein was secreted from the cells because of its N-terminal signal peptide, but because of the PEP-Cterm signal it may have been anchored to the outer membrane of the cells and removed by centrifugation of cell debris.

The predicted model of nIBP appears reasonable, as it positions multiple serine and threonine residues with outward-directed side chains in orderly rows on the b side of the molecule that is considered the ice-binding site in numerous other IBPs whose structures have been determined by X-ray diffraction (8, 9, 21–23, 30–33). In addition, the spacing between the coils (∼4.7 Å) is close to the repeat distance along the ice *a* axis (4.51 Å) and similar to the repeat distance of 4.75 Å measured in a crystallized fungal DUF3494-type IBP (9).

The identity of nIBP and *S. acidiphila* IBP (69%) is substantially higher than identities previously found for IBPs from different phyla (47 to 60%) (15, 27), as well as higher than Doolittle’s 60% identity criterion for HGT (34). The closeness of nIBP to planctomycete IBPs and its distance from IBPs of a cyanobacterial relative as well as its “planctomycete-like” GC content raise the possibility that nIBP was acquired from a planctomycete donor. In fact, the McMurdo Dry Valleys is a possible site of gene transfer, as planctomycetes were reported to comprise about 2% of the microbial community in the black *Nostoc* mats (17). However, it should be pointed out that other gene donors with higher identities might be found in the future.

While IBP genes appear to be relatively rare in cyanobacteria, IBP activity has also been observed in *Phormidium*-like (16) and *Oscillatoria*-like (J. A. Raymond, unpublished data) cyanobacteria from the McMurdo Dry Valleys. If these activities can be attributed to cyanobacterial genes (rather than epiphytic bacterial genes), these genes could provide valuable clues to the origin of cyanobacterial IBPs and whether they form a mono- or polyphyletic family. Furthermore, nIBP is one of the first PEP-Cterm proteins to have a known function and thus could be useful as a model for understanding the roles of these proteins. Finally, for nIBP, further studies are needed to determine whether it is in fact attached to the outer membrane and whether it is glycosylated, as is suspected to be the case for other PEP-Cterm proteins (28). As a glycosylated protein, it may contribute to an exopolysaccharide matrix that is thought to protect the cells from desiccation and other stresses.

## MATERIALS AND METHODS

### Samples.

*Nostoc* samples were collected on 28 January 2000. The collection site is described in Results. The samples were shipped to the University of Nevada Las Vegas (UNLV) and stored at −25°C. *Nostoc* ANT.L52B.8 was obtained from the BCCM Cyanobacteria Collection in Liège, Belgium.

### Sequencing.

An environmental sample of desiccated *Nostoc*, after storage at −25°C for 16 years, was ground in liquid nitrogen. DNA was extracted with a Nucleospin kit (Macherey-Nagel) using lysis buffer PL2 and sent to the Nevada Genomics Center (NGC) at the University of Nevada Reno for sequencing. A library was prepared using the Ion Xpress Plus fragment library kit (Life Technologies) and sequenced on an Ion Torrent Proton platform using Hi-Q chemistry and a P1 chip (Life Technologies), yielding about 86.1 million reads with an average length of 181 bp. The reads were assembled into 877,292 contigs (average length, 702 bp) with CLC Genomics Workbench, with a total length of about 616 million bp.

### 16S analysis of the metagenome.

*Nostoc*-like 16S rRNA sequences were abundant in the metagenome. They were overwhelmingly of a single type, allowing the complete sequence to be assembled (GenBank accession no. MN081603). The species was designated *Nostoc* sp. HG1, after its location near the Hughes Glacier. To identify the most prominent organisms in the sample, we first searched the 86.1 million reads for matches to HG1 16S rRNA with E values of <1e−30 and identities of >97%, obtaining 28,869 hits. These hits were classified as HG1 16S reads. We then searched the files for reads that matched universal bacterial 16S rRNA primers 8F (5′-AGAGTTTGATCCTGGCTCAG-3′, 518R (5′-GTATTACCGCGGCTGCTGG-3′), and 1492R (5′-CGGTTACCTTGTTACGACTT-3′) that matched at least 18 of the 19 or 20 nucleotides in the primers, using a word size of 7. From these hits, we subtracted reads that had been classified as HG1 reads, yielding 11,502 unique non-HG1 reads. These reads were BLAST searched against NCBI’s rRNA_typestrains/prokaryotic_16S_ribosomal_RNA database for matches with E values of <1e−30, yielding 6,922 hits. The hits were downloaded into an XML file and sorted by species using the blastn output “hit_def.” The total hits (28,869 + 6922 = 35,791 reads) were analyzed by species using a pivot table in Excel and displayed in a pie chart, also in Excel.

### IBP gene search.

The 616-Mb contig file was searched with tblastn using 270 DUF3494 domains from representative IBP sequences from bacteria, fungi, and algae as queries, using an E value cutoff of 1e−10. The contig file was also searched for other known IBPs, including ice-nucleating proteins and plant antifreeze proteins.

### Identification of PEP-Cterm proteins.

The proteomes of selected *Nostoc* species, including the closely related *Nostoc* sp. “Peltigera membranacea cyanobiont” N6, were screened for the annotation “PEP-Cterm.” These proteins were used as queries for tblastn searches of the *Nostoc* sp. HG1 contig file. Hits that did not have a PEP-Cterm signal and that did not have a closest match that belonged to the genus *Nostoc* were discarded. The remaining proteins were regarded as *Nostoc* sp. HG1 proteins. A logo diagram of the aligned C-terminal sequences was generated by WebLogo (https://weblogo.berkeley.edu/logo.cgi). Gaps in the logo diagram, which were due to weak signals, were deleted. One of the proteins with an unusually long PEP-Cterm signal was discarded from the analysis.

### Production of recombinant IBPs.

The detailed methods are given in the supplemental material. Briefly, the *Nostoc* IBP (nIBP) sequence minus its N-terminal signal peptide and containing its PEP-Cterm signal (rnIBP [Fig. S1A]) was optimized for expression in E. coli, chemically synthetized, and cloned (GenScript, Piscataway, NJ) in frame with a C-terminal His tag into a pET30a vector (Novagen-Merck, Darmstadt, Germany). Production assays of rnIBP were performed under different conditions (see Table S1). The sequence of nIBP lacking the PEP-Cterm domain (rnIBPΔPEP) was cloned into a pET-21 [SUMO] vector (Fig. S1B). The fusion protein SUMO-nIBPΔPEP was produced as described previously (35). SUMO protein is intended to improve the solubility of the recombinant protein during expression and is then removed during purification before the activity assays are performed. Recombinant His-tagged proteins were extracted and purified by immobilized-metal affinity chromatography (IMAC) on a nickel/nitrilotriacetic acid (Ni/NTA) agarose column (Jena Bioscience, Jena, Germany) from the soluble fraction of E. coli cells as previously described (36). Samples were desalted, lyophilized, and stored at −20°C. Lyophilized samples of rnIBP and rnIBPΔPEP were sent to UNLV. At UNLV, the samples, which had the appearance of a glass, were suspended in 400 μl of deionized (DI) water or 25 mM phosphate buffer but were poorly soluble. One of the rnIBP samples showed IBP activity after it was treated with 0.1% Triton-X and shaken for 30 min at 37°C in an Eppendorf Thermomixer operating at 700 rpm.

### CD spectroscopy.

Circular dichroism (CD) analyses were carried out on rnIBP and rnIBPΔPEP dissolved in 25 mM sodium phosphate buffer, pH 7, at concentrations of 5 μM. Spectra were recorded with a J-815 spectropolarimeter (Jasco Corp., Easton, MD), using a 0.1-cm-path-length quartz cuvette. Spectra were measured in the range of 190 to 260 nm with 0.2-nm data pitch and 20-nm/min scanning speed. All spectra were corrected for buffer contribution, averaged from two independent acquisitions, and smoothed by using a third-order least square polynomial fit. Thermal denaturation spectra were obtained by measuring the CD signal at 204 nm while progressively heating the sample from 25 to 90°C. Measurements were performed in triplicate with a data pitch of 1°C and a temperature slope of 0.5°C/min.

### Purification and activity of IBPs from environmental samples.

Aliquots of the *Nostoc* sample collected in January 2000 were assayed for IBP activity. To determine whether IBPs were present on the *Nostoc* surface, an ∼100-mg clump of *Nostoc* was rinsed with about 100 ml of DI water. The rinse water was clarified by centrifugation, freeze-dried, and resuspended in about 1 ml of water. Other samples (typically 3 g) were ground with a mortar and pestle with ∼20 ml of DI water and centrifuged at 10,000 rpm for 10 min at 2°C. The pellets were subjected to two more cycles of grinding and centrifugation, yielding a pooled supernatant of about 60 ml. IBPs were semipurified by three cycles of ice-affinity purification, using two methods: salting, freezing, and centrifugation (37) and ice shell formation (38). Ice-binding activity was estimated by observing irregularities in the surface of a growing ice seed crystal submerged in the supernatant (37).

### Mass spectrometry analyses.

Freeze-dried, ice-affinity purified material was sent to the Medical University of South Carolina (MUSC) and later to the College of Charleston (Charleston, SC), for liquid chromatography/mass spectrometry (LC/MS) analysis of tryptic peptides. Approximately 20 μg of total protein (as estimated from gel electrophoresis) was digested overnight at 37°C with trypsin gold (1:20; Promega) or GluC (1:10; Promega). Peptides were separated using two LC/MS platforms, an Eksigent nano-LC coupled to a SCIEX 5600 with a nanospray source and a Dionex Ultimate 3000 nano-LC coupled to an Orbitrap Fusion Lumos with a nanospray source at Hollings Marine Laboratory (Charleston, SC). Data were acquired from the Eksigent platform as described previously (39) and from the Dionex platform as described previously (40). Masses of the tryptic peptides were compared with predicted tryptic peptides in the *Nostoc* contig file translated in all six frames using MASCOT and MS-GF+ search engines. Searches included standard variable modifications and carbamidomethyl-fixed modifications. The search parameter Enzyme was set to trypsin or semitrypsin for data from trypsin digests or GluC(DE) for GluC endopeptidase digests. Error-tolerant searches were conducted to look for modified peptides not otherwise included.

### 3D structure prediction.

The 3D structure of the DUF3494 domain of nIBP was predicted with Swiss Model (44) (http://swissmodel.expasy.org) using the structure of *Efc*IBP (PDB code 6EIO; sequence identity, 52% [21]) as the template. The free energy of the model was then minimized (from −65,568 kJ/mol to −93392 kJ/mol) by the Yasara minimization server (http://www.yasara.org/minimizationserver.htm) (41) and viewed with Yasara v. 19.7.20. Stereoviews of the molecule were obtained by rotating it around the *y* axis by 3°. Distance measurements were made with the Yasara distance tool.

Signal peptides were predicted with SignalP http://www.cbs.dtu.dk/services/SignalP-3.0/(42). DUF3494 domains were identified with NCBI’s conserved domain database https://www.ncbi.nlm.nih.gov/Structure/cdd/wrpsb.cgi. Potential N- and O-glycosylation sites were predicted with GlycoPP (http://crdd.osdd.net/raghava/glycopp/) (43).

### Data availability.

The whole-genome shotgun project has been deposited at DDBJ/ENA/GenBank under the accession no. VJOX00000000.

## Supplementary Material

Supplemental file 1

## References

[1] Davies PL 2014 Ice-binding proteins: a remarkable diversity of structures for stopping and starting ice growth. Trends Biochem Sci 39:548–555. doi:10.1016/j.tibs.2014.09.005. 25440715

[2] Raymond JA, Remias D 2019 Ice-binding proteins in a Chrysophycean snow alga: acquisition of an essential gene by horizontal gene transfer. Front Microbiol 10:2697. doi:10.3389/fmicb.2019.02697. 31849866 PMC6892780

[3] Duman JG 2015 Animal ice-binding (antifreeze) proteins and glycolipids: an overview with emphasis on physiological function. J Exp Biol 218:1846–1855. doi:10.1242/jeb.116905. 26085662

[4] Bayer-Giraldi M, Weikusat I, Besir H, Dieckmann G 2011 Characterization of an antifreeze protein from the polar diatom Fragilariopsis cylindrus and its relevance in sea ice. Cryobiology 63:210–219. doi:10.1016/j.cryobiol.2011.08.006. 21906587

[5] Raymond JA 2011 Algal ice-binding proteins change the structure of sea ice. Proc Natl Acad Sci U S A 108:E198. doi:10.1073/pnas.1106288108. 21636791 PMC3116383

[6] Raymond JA, Christner BC, Schuster SC 2008 A bacterial ice-binding protein from the Vostok ice core. Extremophiles 12:713–717. doi:10.1007/s00792-008-0178-2. 18622572

[7] Guo SQ, Langelaan DN, Phippen SW, Smith SP, Voets IK, Davies PL 2018 Conserved structural features anchor biofilm-associated RTX-adhesins to the outer membrane of bacteria. FEBS J 285:1812–1826. doi:10.1111/febs.14441. 29575515

[8] Vance TDR, Graham LA, Davies PL 2018 An ice-binding and tandem beta-sandwich domain-containing protein in Shewanella frigidimarina is a potential new type of ice adhesin. FEBS J 285:1511–1527. doi:10.1111/febs.14424. 29498209

[9] Kondo H, Hanada Y, Sugimoto H, Hoshino T, Garnham CP, Davies PL, Tsuda S 2012 Ice-binding site of snow mold fungus antifreeze protein deviates from structural regularity and high conservation. Proc Natl Acad Sci U S A 109:9360–9365. doi:10.1073/pnas.1121607109. 22645341 PMC3386094

[10] Vance TDR, Bayer-Giraldi M, Davies PL, Mangiagalli M 2019 Ice-binding proteins and the ‘domain of unknown function’ 3494 family. FEBS J 286:855–873. doi:10.1111/febs.14764. 30680879

[11] Raymond JA, Janech MG, Fritsen CH 2009 Novel ice-binding proteins from a psychrophilic antarctic alga (Chlamydomonadaceae, Chlorophyceae). J Phycol 45:130–136. doi:10.1111/j.1529-8817.2008.00623.x. 27033652

[12] Jung W, Campbell RL, Gwak Y, Kim JI, Davies PL, Jin E 2016 New cysteine-rich ice-binding protein secreted from Antarctic microalga, *Chloromonas* sp. PLoS One 11:e0154056. doi:10.1371/journal.pone.0154056. 27097164 PMC4838330

[13] Cho SM, Kim S, Cho H, Lee H, Lee JH, Lee H, Park H, Kang S, Choi H-G, Lee J 2019 Type II ice-binding proteins isolated from an arctic microalga are similar to adhesin-like proteins and increase freezing tolerance in transgenic plants. Plant Cell Physiol 60:2744–2757. doi:10.1093/pcp/pcz162. 31418793

[14] Arai T, Fukami D, Hoshino T, Kondo H, Tsuda S 2019 Ice-binding proteins from the fungus Antarctomyces psychrotrophicus possibly originate from two different bacteria through horizontal gene transfer. FEBS J 286:946–962. doi:10.1111/febs.14725. 30548092

[15] Raymond JA, Kim HJ 2012 Possible role of horizontal gene transfer in the colonization of sea ice by algae. PLoS One 7:e35968. doi:10.1371/journal.pone.0035968. 22567121 PMC3342323

[16] Raymond JA, Fritsen CH 2000 Ice-active substances associated with Antarctic freshwater and terrestrial photosynthetic organisms. Antarct Sci 12:418–424. doi:10.1017/S0954102000000493.

[17] Van Horn DJ, Wolf CR, Colman DR, Jiang X, Kohler TJ, McKnight DM, Stanish LF, Yazzie T, Takacs-Vesbach CD 2016 Patterns of bacterial biodiversity in the glacial meltwater streams of the McMurdo Dry Valleys, Antarctica. FEMS Microbiol Ecol 92:fiw148. doi:10.1093/femsec/fiw148. 27495241 PMC5975864

[18] Taton A, Grubisic S, Balthasart P, Hodgson DA, Laybourn-Parry J, Wilmotte A 2006 Biogeographical distribution and ecological ranges of benthic cyanobacteria in East Antarctic lakes. FEMS Microbiol Ecol 57:272–289. doi:10.1111/j.1574-6941.2006.00110.x. 16867145

[19] Zúñiga C, Leiva D, Ramírez-Fernández L, Carú M, Yahr R, Orlando J 2015 Phylogenetic diversity of *Peltigera* cyanolichens and their photobionts in Southern Chile and Antarctica. Microbes Environ 30:172–179. doi:10.1264/jsme2.ME14156. 25925273 PMC4462928

[20] Gagunashvili AN, Andrésson ÓS 2018 Distinctive characters of Nostoc genomes in cyanolichens. BMC Genomics 19:434. doi:10.1186/s12864-018-4743-5. 29866043 PMC5987646

[21] Mangiagalli M, Sarusi G, Kaleda A, Bar Dolev M, Nardone V, Vena VF, Braslavsky I, Lotti M, Nardini M 2018 Structure of a bacterial ice binding protein with two faces of interaction with ice. FEBS J 285:1653–1666. doi:10.1111/febs.14434. 29533528

[22] Yamauchi A, Arai T, Kondo H, Sasaki YC, Tsuda S 2020 An ice-binding protein from an Antarctic ascomycete is fine-tuned to bind to specific water molecules located in the ice prism planes. Biomolecules 10:759. doi:10.3390/biom10050759. PMC7277481 32414092

[23] Do H, Kim SJ, Kim HJ, Lee JH 2014 Structure-based characterization and antifreeze properties of a hyperactive ice-binding protein from the Antarctic bacterium *Flavobacterium frigoris* PS1. Acta Crystallogr D Biol Crystallogr 70:1061–1073. doi:10.1107/S1399004714000996. 24699650

[24] Guo M, Han X, Jin T, Zhou L, Yang J, Li Z, Chen J, Geng B, Zou Y, Wan D, Li D, Dai W, Wang H, Chen Y, Ni P, Fang C, Yang R 2012 Genome sequences of three species in the family Planctomycetaceae. J Bacteriol 194:3740–3741. doi:10.1128/JB.00639-12. 22740668 PMC3393480

[25] Di Rienzi SC, Sharon I, Wrighton KC, Koren O, Hug LA, Thomas BC, Goodrich JK, Bell JT, Spector TD, Banfield JF, Ley RE 2013 The human gut and groundwater harbor non-photosynthetic bacteria belonging to a new candidate phylum sibling to Cyanobacteria. Elife 2:e01102. doi:10.7554/eLife.01102. 24137540 PMC3787301

[26] Raymond JA 2016 Dependence on epiphytic bacteria for freezing protection in an Antarctic moss, Bryum argenteum. Environ Microbiol Rep 8:14–19. doi:10.1111/1758-2229.12337. 26417678

[27] Raymond JA 2014 The ice-binding proteins of a snow alga, *Chloromonas brevispina*: probable acquisition by horizontal gene transfer. Extremophiles 18:987–994. doi:10.1007/s00792-014-0668-3. 25081506

[28] Haft DH, Paulsen IT, Ward N, Selengut JD 2006 Exopolysaccharide-associated protein sorting in environmental organisms: the PEP-CTERM/EpsH system. Application of a novel phylogenetic profiling heuristic. BMC Biol 4:29. doi:10.1186/1741-7007-4-29. 16930487 PMC1569441

[29] Tamaru Y, Takani Y, Yoshida T, Sakamoto T 2005 Crucial role of extracellular polysaccharides in desiccation and freezing tolerance in the terrestrial cyanobacterium Nostoc commune. Appl Environ Microbiol 71:7327–7333. doi:10.1128/AEM.71.11.7327-7333.2005. 16269775 PMC1287664

[30] Cheng J, Hanada Y, Miura A, Tsuda S, Kondo H 2016 Hydrophobic ice-binding sites confer hyperactivity of an antifreeze protein from a snow mold fungus. Biochem J 473:4011–4026. doi:10.1042/BCJ20160543. 27613857

[31] Hanada Y, Nishimiya Y, Miura A, Tsuda S, Kondo H 2014 Hyperactive antifreeze protein from an Antarctic sea ice bacterium Colwellia sp. has a compound ice-binding site without repetitive sequences. FEBS J 281:3576–3590. doi:10.1111/febs.12878. 24938370

[32] Kondo H, Mochizuki K, Bayer-Giraldi M 2018 Multiple binding modes of a moderate ice-binding protein from a polar microalga. Phys Chem Chem Phys 20:25295–25303. doi:10.1039/c8cp04727h. 30255887

[33] Lee JH, Park AK, Do H, Park KS, Moh SH, Chi YM, Kim HJ 2012 Structural basis for antifreeze activity of ice-binding protein from arctic yeast. J Biol Chem 287:11460–11468. doi:10.1074/jbc.M111.331835. 22303017 PMC3322824

[34] Doolittle RF 2002 Gene transfers between distantly related organisms, p 269–275. *In* Syvanen M, Kado CI (ed), Horizontal gene transfer. Academic Press, San Diego, CA. doi:10.1016/B978-012680126-2/50031-1.

[35] de Marco A, Vigh L, Diamant S, Goloubinoff P 2005 Native folding of aggregation-prone recombinant proteins in Escherichia coli by osmolytes, plasmid- or benzyl alcohol-overexpressed molecular chaperones. Cell Stress Chaperones 10:329–339. doi:10.1379/csc-139r.1. 16333986 PMC1283876

[36] Mangiagalli M, Bar-Dolev M, Tedesco P, Natalello A, Kaleda A, Brocca S, de Pascale D, Pucciarelli S, Miceli C, Braslavsky I, Lotti M 2017 Cryo-protective effect of an ice-binding protein derived from Antarctic bacteria. FEBS J 284:163–177. doi:10.1111/febs.13965. 27860412

[37] Raymond JA, Fritsen CH 2001 Semipurification and ice recrystallization inhibition activity of ice-active substances associated with Antarctic photosynthetic organisms. Cryobiology 43:63–70. doi:10.1006/cryo.2001.2341. 11812052

[38] Marshall CJ, Basu K, Davies PL 2016 Ice-shell purification of ice-binding proteins. Cryobiology 72:258–263. doi:10.1016/j.cryobiol.2016.03.009. 27025155

[39] Sobolesky P, Parry C, Boxall B, Wells R, Venn-Watson S, Janech MG 2016 Proteomic analysis of non-depleted serum proteins from bottlenose dolphins uncovers a high vanin-1 phenotype. Sci Rep 6:33879. doi:10.1038/srep33879. 27667588 PMC5036180

[40] Neely BA, Prager KC, Bland AM, Fontaine C, Gulland FM, Janech MG 2018 Proteomic Analysis of urine from California sea lions (Zalophus californianus): a resource for urinary biomarker discovery. J Proteome Res 17:3281–3291. doi:10.1021/acs.jproteome.8b00416. 30113852 PMC7607554

[41] Krieger E, Joo K, Lee J, Lee J, Raman S, Thompson J, Tyka M, Baker D, Karplus K 2009 Improving physical realism, stereochemistry, and side-chain accuracy in homology modeling: four approaches that performed well in CASP8. Proteins 77:114–122. doi:10.1002/prot.22570. 19768677 PMC2922016

[42] Bendtsen JD, Nielsen H, von Heijne G, Brunak S 2004 Improved prediction of signal peptides: SignalP 3.0. J Mol Biol 340:783–795. doi:10.1016/j.jmb.2004.05.028. 15223320

[43] Chauhan JS, Bhat AH, Raghava GPS, Rao A 2012 GlycoPP: a webserver for prediction of N- and O-gycosites in prokaryotic protein sequences. PLoS One 7:e40155. doi:10.1371/journal.pone.0040155. 22808107 PMC3392279

[44] Arnold K, Bordoli L, Kopp J, Schwede T 2006 The SWISS-MODEL workspace: a web-based environment for protein structure homology modelling. Bioinformatics 22:195–201. doi:10.1093/bioinformatics/bti770. 16301204

