# Supplementary material for: Ice-Binding Proteins Associated with an Antarctic Cyanobacterium, Nostoc sp. HG1
Source: Appl Environ Microbiol. 2021 Jan 4;87(2):e02499-20. doi: 10.1128/AEM.02499-20 (PMC7783341; doi:10.1128/AEM.02499-20)
Supplement: Supplemental file 1 [file AEM.02499-20-s0001.pdf]

Supplementary materials

Production of rnIBP

The gene encoding nIBP (Fig. S1A) was cloned in pET-30a vector between *Nde*I and *Xho*I sites. Transformed *E. coli* cells were cultured under different conditions (Table S1). Cells were harvested by centrifugation at 1600 x g at 4°C and re-suspended in lysis buffer (50 mM sodium phosphate pH 8.0, 300 mM NaCl, and 10 mM imidazole) and lysed using a cell disruptor (Constant Systems Ltd) at 25,000 psi. Insoluble proteins were pelleted by centrifugation (15 min at 15,000 x g, 4°C). Most preparations were unsuccessful due to formation of inclusion bodies. Recombinant nIBP was purified by immobilized-metal affinity chromatography (IMAC) on an Ni/NTA agarose column (Jena Bioscience, Jena, Germany). Samples containing the highest protein concentrations were desalted on a PD10 desalting column (GE Healthcare, Little Chalfont, UK) and then equilibrated to ammonium acetate buffer (10 mM, pH 7).
Protein concentration was determined by the Bradford protein assay (Bio-Rad), using bovine serum albumin as the standard. SDS/PAGE was on 14% acrylamide gels (Laemmli, 1970) stained with Coomassie dye (Bio-Rad, Hercules, CA, USA) after electrophoresis. Broad-range, pre-stained molecular-mass markers (GeneSpin, Milan, Italy) were used as standards.
A small amount of purified rnIBP (50 μg per liter of culture) was obtained at 20°C in Zym-5052 medium.

Production of rnIBPΔPEP

The gene encoding nIBPΔPEP was cloned downstream of His-tagged SUMO gene in the vector pET-21a [SUMO], between *BamH*I and *Xho*I sites to obtain pET-21 [SUMO-nIBPΔPEP] vector. The sequence and architecture of SUMO-nIBPΔPEP are shown in Fig. S1B. The expression vector pET-28[SenP2] (Reverter and Lima, 2004) was kindly provided by David Reverter (Universitat Autònoma de Barcelona). The fusion protein SUMO-nIBPΔPEP was produced as described (De Marco et al., 2005). Briefly, cultures were grown in LB containing 100 mg/mL ampicillin at 37°C until OD_{600} reached ~ 0.6–0.8. Benzyl alcohol was then added to a final concentration of 10 mM. After 30 minutes, expression was induced with 0.1 mM IPTG. The cells were incubated at 20°C for 16 h. Cells were extracted and purified as described above. SUMO protein was cleaved by incubating the recombinant protein with SenP2 protease (1:100 w/w), releasing His-tagged SUMO. The digest was applied to a second IMAC column, in which the recombinant protein eluted with the flow-through and the His-tagged SUMO and SenP2 proteins were retained on the column. nIBPΔPEP in the flow-through fraction was desalted as described above. The samples were lyophilized in a freeze-dryer (Heto FD1.0, Gemini BV, Apeldoorn, the Netherlands) and stored at -20°C. The fusion protein SUMO-nIBPΔPEP was produced with a total yield, after removing SUMO, of 150 μg pure protein per liter of culture. The total yield of the fusion protein nIBPΔPEP after removing SUMO was 150 μg pure protein per liter of culture.

Table S1. Production conditions of rnIBP

Medium	LB		Zym-5052	
Host	Arctic express (DE3)[1]	BL21 (DE3)	BL21 (DE3)	
Temperature	15°C	20°C	25°C	20°C
Inducer	IPTG	IPTG	Lactose	Lactose
Induction time (h)	16	8	18	18
Production yield	n.a.	n.a.	n.a.	50 μg/L

[1] Expression carried out by GenScript (Piscataway, NJ)

Table S2. Bacterial IBPs found in the *Nostoc* sp. HG1 metagenome. The IBP sequences in these contigs are located here.

Contig	Signal Peptide?	No. DUF3494 domains	Closest match	
			Species	Acc. number
105	y	1	*Flavisolibacter*	WP_139256525
3866	y	2	*Bryum bacterium*	ALG05197
9736	y	1	*Spirosoma*	WP_142771422
20932	y	2	*Flavobacterium*	WP_121314189
21986	?	1	*Flavobacterium*	WP_091433241
23325	y	1	*Runella*	WP_114070612
51291	y	1	*Burkholderiales*	OYT90918
71676	y	1	*Bryum* epiphyte	ALG05180
78691	y	1	*Hymenobacter*	WP_081867695
82099	y	1	*Aequorivita*	WP_146848183
90703	?	1	*Chryseobacterium*	WP_133439926
95672	y	1	*Hymenobacter*	WP_081867695
116446	y	2	*Bryum* epiphyte	ALG05197
137179	y	2	*Flavobacterium*	WP_121314189
141220	y	2	*Bacteroidetes*	TND00507
142853	y	1	uncultured	AHG59377
145048	y	1	*Larkinella*	WP_124902832
185788	y	1	*Cytophagales*	HCM76882
188129	y	1	*Spirosoma*	WP_020607028
193142	y	1	*Nostoc*	WP_099100461
206564	y	1	*Flavobacterium*	WP_091433286
212299	y	2	*Flavobacterium*	WP_121314189
224239	y	1	*Bryum* epiphyte	ALG05163
226551	y	1	*Sulfuriferula*	WP_147070280
321469	y	1	*Litoribacter*	WP_143961148

Table S3. *Nostoc* species that are closely related to *Nostoc* sp. HG1 and that are symbionts of the lichen *Peltigera*.

Species	Country	IBP accession	Reference	Genome scaffold	*Nostoc* sp. HG1 IBP[1]	
					ID	Sim
Nostoc sp. 'Peltigera malacea cyanobiont' DB3992	Canada	WP_ 099100461	Gagunashvili and Andresson 2018	NZ_NSHF01000067	83	89
Nostoc punctiforme NIES-2108	Japan	RCJ34851		LXQE01000154	83	86
Nostoc sp. 'Peltigera membranacea cyanobiont' N6	Iceland	WP_ 104901965	Gagunashvili and Andresson 2018	NZ_CP026681	80	86

[1]Identity and similarity percents to *Nostoc* HG1 IBP DUF3494 domain.

Table S4. Distance between coils on the ice-binding site of the model structure of *Nostoc* IBP

Residue/side chain atom[1]		Number of coils	Distance (A)	
From	To		Total	Aveage
Thr67 CA	Gln130 CA	6	28.406	4.73
Thr67 HG1	Gln130 OE1	6	25.961	4.32
Thr67 HG1	Thr156 HG1	5	23.247	4.65
Ser65 CA	Ser128 CA	6	29.584	4.93
Ser65 HG	Ser128 HG	6	27.417	4.57

[1]CA, alpha C; HG1 and HG, H on OH group of side chain; OE1, O on side chain.

Table S5. *Nostoc* sp. HG1 exosortase system associated with PEP-Cterm proteins. The genes are on contig 279 (13.7 kb) in the indicated order. The gene sequences are available here.

Gene order	Gene	Length (bp)
1	ictB putative inorganic carbon transporter	1428
2	GAF domain-containing sensor histidine kinase	1554
3	DEGTDnrJ/Eryc1/strs family aminotransferase	1194
4	EPSH	897
5	cyanoexosortase B system-associated protein	723
6	polysaccharide export protein	1356
7	polysaccharide biosynthesis tyrosine autokinase	2175
8	rhomboid family intramembrane serine protease	618
9	rhomboid family intramembrane serine protease	456

Supplementary figures

Fig. S1. Sequences and architecture of rnIBP (A) and SUMO-rnIBPΔPEP (B). Yellow, DUF3494 domain; blue, PEP-Cterm signal, green, SUMO protein; His-tag is in bold.

Fig. S2. Additional views of Nostoc IBP structure. A) The 3D structure of the nIBP was modelled using *Efc*IBP (PDB: 6EIO) as a template. β strands belonging to the a, b, and c faces are in green, yellow, and cyan, respectively. The α-helix is in red and the 3_{10} helix in magenta. B) Ice binding site prediction. Sequence alignment of the IBS from *Efc*IBP (Mangiagalli et al., 2018), *Anp*IBP (Yamauchi et al., 2020) and nIBP. Outward-pointing residues and inward-pointing residues are indicated in capital and small letters, respectively. Residues involved in ice binding in *Efc*IBP and *Anp*IBP are in bold.

References

De Marco, A, Vigh, L., Diamant, S, and Goloubinoff, P. 2005. Native folding of aggregation-prone recombinant proteins in *Escherichia coli* by osmolytes, plasmid-or benzyl alcohol–overexpressed molecular chaperones. Cell Stress & Chaperones 10:329.

Laemmli, U.K. 1970. Cleavage of structural proteins during the assembly of the head of bacteriophage T4. Nature 227:680-685.

Mangiagalli M, Sarusi G, Kaleda A, Bar Dolev M, Nardone V, Vena VF, Braslavsky I, Lotti M, Nardini M. 2018. Structure of a bacterial ice-binding protein with two faces of interaction with ice. FEBS J 285:1653–1666.

Reverter, D, and Lima, CD. 2004. A Basis for SUMO protease specificity provided by analysis of human Senp2 and a Senp2-SUMO complex. Structure 12:1519-1531.

Yamauchi A, Arai T, Kondo H, Sasaki YC, Tsuda S. 2020. An ice-binding protein from an Antarctic ascomycete is fine-tuned to bind to specific water molecules located in the ice prism planes. Biomolecules 10:759.
